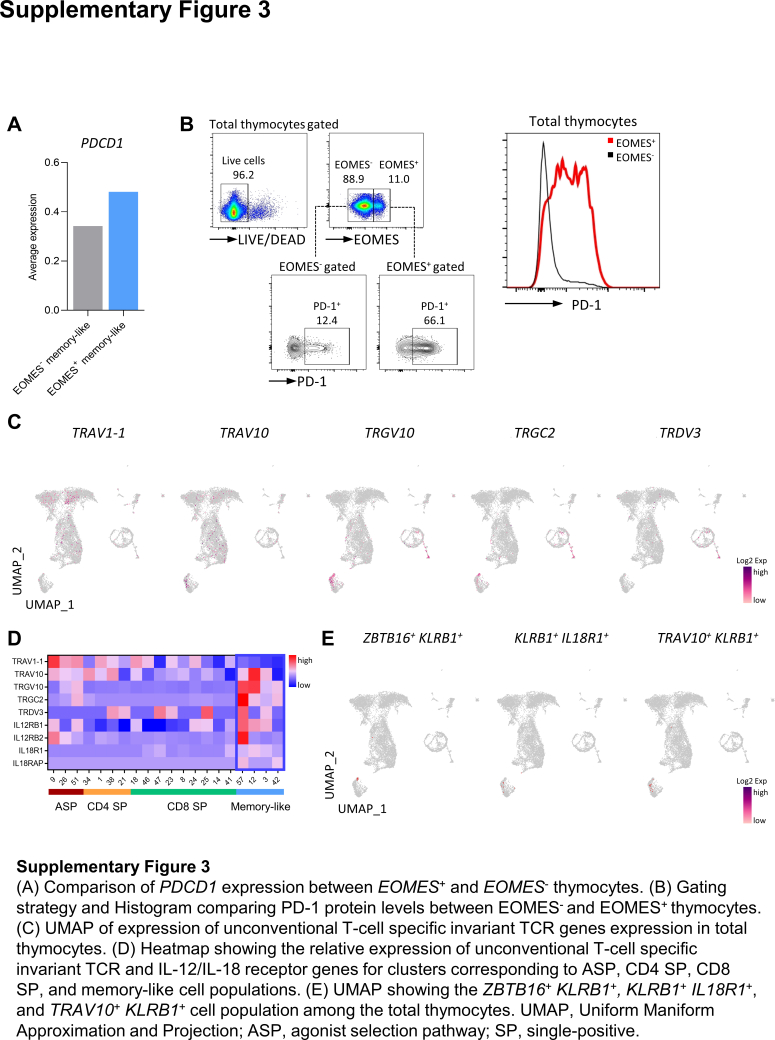# Developmental trajectory of unconventional T cells of the cynomolgus macaque thymus

**DOI:** 10.1016/j.heliyon.2024.e39736

**Published:** 2024-10-23

**Authors:** Sung Min Choi, Kyeong Cheon Jung, Jae Il Lee

**Affiliations:** a Graduate Course of Translational Medicine, Seoul National University College of Medicine, Seoul, 03080, Republic of Korea; b Transplantation Research Institute, Seoul National University College of Medicine, Seoul, 03080, Republic of Korea; c Department of Pathology, Seoul National University College of Medicine, Seoul, 03080, Republic of Korea; d Integrated Major in Innovative Medical Science, Seoul National University Graduate School, Seoul, 03080, Republic of Korea; e Department of Medicine, Seoul National University College of Medicine, Seoul, 03080, Republic of Korea

**Keywords:** Single-cell RNA sequencing, Agonist-selected T cells, Memory-like T cells, T-cell receptor signaling, *PDCD1*, *EOMES*

## Abstract

As nonhuman primates are immunologically the closest model to humans, a comprehensive understanding of T-cell development in these species is crucial. However, the differentiation pathways in which thymocytes participate, along with their heterogeneity, remain poorly characterized. Using single-cell RNA sequencing, we thoroughly profiled the development of various T-cell lineages in the juvenile cynomolgus monkey thymus, identifying and characterizing 12 distinct thymic cell states or types. Interestingly, we identified two unexpected cell types, an agonist-selected and a memory-like cell population. The agonist-selected cell population expressed genes associated with strong TCR signaling, such as *PDCD1*, *CD5*, *NFKBID*, *NFATC1*, *BCL2L11*, and *NR4A1* but exhibiting significantly higher *PDCD1* expression compared with cells following the conventional developmental pathway. Additionally, we identified a substantial number of memory-like cell populations characterized by high *CXCR3* and *EOMES* expression. Notably, this population also highly expressed the effector-associated markers, *GZMK*, *NKG7*, and *GNLY*, as well as the innate cell-associated markers, *ZBTB16*, *TYROBP*, *KLRB1*, *KLRC1*, and *NCR3*. The *EOMES* ^+^ memory-like cell population expressed highly *PDCD1*, indicating the presence of an agonist-selection footprint. Our findings provide insights into the agonist-selection pathway that allows self-reactive thymocytes to survive thymic selections and differentiate into various unconventional T-cell lineages.

## 1 Introduction

The thymus plays a pivotal role in generating diverse T-cell lineages that are able to mount effective immune responses against potential pathogens and in maintaining the self-tolerance of the immune system towards host antigens. Thymocytes first undergo recombination to determine γδ or αβ T cell lineage at the CD4^−^CD8^−^double-negative (DN) stage, followed by random recombination to complete the αβ T cell receptor (TCR) gene at the CD4^+^CD8^+^ double-positive (DP) stage [1,2]. A checkpoint determines whether successful rearrangement of TCR chains has occurred through the positive selection, and the strength of TCR signaling determines the lineage outcome [3,4]. Most mature αβTCR^+^ cells generated by conventional selection display low binding affinity for self-peptide major histocompatibility complexes (MHCs) and exit the thymus as naïve CD4 or CD8 single-positive (SP) T cells [4]. Conversely, immature thymocytes with rearranged TCRs that strongly react with self-peptide MHCs can enter the conventional naïve T-cell pool and potentially lead to severe autoimmunity [5], unless they are eliminated by negative selection.

However, not all autoreactive immature thymocytes are eliminated by conventional negative selection. Some of them are preserved and adopt distinct functional fates by developing along the agonist-selection pathway [6,7]. Agonist-selected unconventional T cells are thought to have acquire diverse functions in the immune system, such as Foxp3^+^ regulatory T (Treg) cells [8], invariant natural killer T cells (iNKT) [9], mucosal-associated invariant T cells (MAIT) [10], TCRαβ^+^CD8αα^+^ intestinal intraepithelial lymphocytes (IELs) [11], and natural Th17 [12] cells. These lineages require relatively strong and continuous TCR signals for their development [13]. Nevertheless, self-reactive thymocytes that survive negative selection and mature into unconventional T-cell lineages are considered divergent or predetermined from thymic progenitors with a naïve phenotype. Recent studies have utilized single-cell analysis to define the developmental trajectories of mouse and human αβ thymocytes [[14], [15], [16]]. Additionally, the heterogeneity of thymic progenitors and unconventional T-cell lineages was studied in the human and mouse thymus by single-cell RNA sequencing [17,18].

However, when and how self-reactive thymocytes with high-affinity TCR:pMHC interactions survive apoptosis through negative selection to generate various unconventional T-cell lineages that bridge innate and adaptive immunity remain ill-defined. Previously, we identified innate-like T cells characterized by *ZBTB16* or *EOMES* expression, which constitute innate-like cell marker genes, in peripheral CD4/CD8 DP T cells [19,20]. Although how these cell populations develop in the thymus remains unclear, the fact that various unconventional T-cell lineages do develop via alternative pathways indicates that progenitor cells are present. To date, the fate and overall developmental trajectory of thymocytes are being elucidated; yet many aspects of T-cell development remain unclear. The primary focus of the study was to determine whether monkey thymocytes could recapitulate the selection pathways observed in humans, and whether T-cell differentiation via agonist selection is associated with strong TCR signaling and discernible during thymocyte maturation. In this study, we aimed to characterize the agonist-selection pathway of self-reactive thymocytes and the development of various unconventional T-cell lineages using single-cell RNA sequencing. Our objective is to provide a comprehensive map of T-cell composition and fate in the cynomolgus monkey, a widely used nonhuman primate model in immunology research, noted for having unconventional T cell pools most similar to those in humans.

## 2 Materials and methods

### 2.1 Subjects

The thymus sample for single-cell RNA sequencing was obtained from a healthy male cynomolgus monkey (*Macaca fascicularis*) aged 2 years. The animal was cared for in strict accordance with the National Institutes of Health Guide for the Care and Use of Laboratory Animals. The study was approved by the local Institutional Animal Care and Use Committee (IACUC) of Seoul National University Hospital (IACUC number: 21-0297-C1A0). All experiments were performed in accordance with the relevant guidelines and regulations and the ARRIVE guidelines.

### 2.2 Cell preparation for single-cell RNA sequencing

The monkey was first deeply anesthetized by an intramuscular injection of ketamine (10 mg/kg, Yuhan, Korea) and an intravenous injection of sodium pentobarbital (25 mg/kg, Hanlim Pharm. Co. Ltd, Korea), and subsequently euthanized by exsanguination. Thymic tissues were processed immediately after isolation, being minced into a single-cell suspension using a 70-μm cell strainer and a syringe plunger. The procedure was repeated until the tissue was completely dissociated. The dissociated cells were then resuspended in RPMI 1640 (Biowest, Nuaillé, France) supplemented with 10 % fetal bovine serum (FBS; Biowest). After isolation, cells were immediately used for single-cell RNA sequencing, and the flow cytometric results obtained using the generated cell suspension were considered representative.

### 2.3 Library construction and sequencing

Libraries were prepared using the Chromium controller according to the 10 × Chromium Next GEM Single Cell 5′ Reagent Kit v2 user guide (CG000331). Briefly, cell suspensions were diluted in nuclease-free water to achieve a target cell count of 10,000. The cell suspension was then mixed with master mix and loaded, along with Single-Cell 5′ Gel Beads and Partitioning Oil, into a Next GEM Chip K. RNA transcripts from single cells were uniquely barcoded and reverse-transcribed within droplets. Next, cDNA molecules were pooled and enriched by PCR using 50 ng of cDNA and 14 amplification cycles. To generate a 5′ Gene Expression Library, the cDNA pool underwent an end-repair process (i.e., the addition of a single deoxyadenosine), followed by ligation of the adapters. The products were then purified and enriched by PCR to create the 5’ Gene Expression Library. The purified libraries were quantified by qPCR according to the qPCR Quantification Protocol Guide (KAPA) and qualified using Agilent Technologies 4200 TapeStation (Agilent Technologies, USA). Finally, the libraries were sequenced using the HiSeq platform (Illumina) according to the read length in the user guide.

### 2.4 Preprocessing and analysis of single-cell RNA-sequencing data

The Cell Ranger v7.0.1 (10 × Genomics) pipeline was used to generate FASTQ files from raw sequencing data for gene expression analysis of 5’ Gene Expression Library data. Briefly, raw BCL files from Illumina HiSeqXten were demultiplexed to generate FASTQ files using “cellranger mkfastq.” These raw FASTQ files were then analyzed using “cellranger count.” The “cellranger count” step included mapping to the *Macaca fascicularis* reference genome (*Macaca_fascicularis_5.0*), measuring gene expression using unique molecular identifiers and cell barcodes, determining cell clusters, and conducting differential gene expression analyses. “Count” was used to take input from multiple sequencing runs on the same library, and cells were grouped into clusters according to gene expression.

### 2.5 Advanced analysis of single-cell RNA-sequencing data

Raw-count 10 × -Genomics matrices were imported into Seurat 4.3.0. Raw counts for all genes expressed in ≥3 cells and from all cells with ≥200 detected genes were used for downstream analysis. We considered that a rare subset of cells with a detectable number of outlier genes identified as potential multiplets, low-quality, or dying cells may still exist; therefore, cells with >5000 genes or mitochondrial counts >20 % were filtered out. After filtering, the count value of 16,469 genes from 10,447 cells was used in the next step. Gene expression values were scaled and normalized for each gene across all integrated cells. Clustering and Uniform Maniform Approximation and Projection (UMAP) analysis were performed based on statistically significant principal components. Then, significant top-cluster markers for every cluster compared with all remaining cells were determined using the Wilcox rank sum test (min.pct = 0.25; logfc.threshold = 0.25; with only the positive ones being filtered). Gene-Enrichment and Functional Annotation analysis for significant gene lists was performed respectively using the g:Profiler tool (https://biit.cs.ut.ee/gprofiler/). Trajectory reconstruction for each data set was performed using Monocle3 1.3.1, which defines a pseudotime measurement across a root cluster group through which the dynamics of gene expression can be examined. All heatmaps, volcano plots, and graphs were generated using GraphPad Prism 8.0.2 (GraphPad Software Inc., San Diego, CA, USA)

### 2.6 Statistical analysis

Loupe Browser software 6.5.0 was used for differential expression analysis and statistical analysis. Loupe Browser utilized the exact negative binomial test to identify significant differences. The p-values calculated using the differential expression analysis feature were adjusted for multiple comparisons using the Benjamini–Hochberg correction.

### 2.7 Flow cytometric analysis

Prior to surface and intracellular staining, Zombie NIR™ Fixable Viability (BioLegend, USA) was used to exclude dead cells. For surface staining, prepared cells were resuspended in staining buffer (PBS, 0.5 % BSA, and 0.5 mM EDTA), and single-cell suspensions were labeled with antibodies for 30 min at 4 °C. After surface staining, the cells were washed and resuspended in staining buffer. For intracellular staining, the surface-stained cells were washed with PBS before fixation and permeabilized using the FoxP3/Transcription factor staining buffer set (eBioscience). Then, intracellular cytokines and/or transcription factors were labeled with antibodies for 30 min at 4 °C. Flow cytometry was performed using an LSRFortessa X-20 or an LSRII cytometer (BD Biosciences). All data were analyzed using the FlowJo software v10 (TreeStar, San Carlos, CA, USA). The following fluorochrome-labeled human monoclonal antibodies were used for flow cytometric analysis: CD3-PerCP-Cy™5.5 (SP34-2, BD Biosciences), CD4-PE/Cyanine7 (OKT4, BioLegend), CD8α-Brilliant Violet 711™ (SK1, BioLegend), PD-1 (CD279)-APC (EH12.2H7, BioLegend), and EOMES-FITC (WD1928, eBioscience).

## 3 Results

### 3.1 T-cell developmental trajectories

To characterize T-cell development in the thymus, we conducted single-cell RNA sequencing using the 10 × Genomics platform on thymocytes from a juvenile cynomolgus monkey. After filtering, we analyzed the expression of 16,469 genes expressed in 10,447 cells and revealed ≥12 distinct thymic cell states or types, along with some undefined non-T cells (Fig. 1A), namely, DN-early and late, γδT, DN–DP transition, DP-early and late, strongly-signaled, agonist-selected, DP–SP transition, CD4^hi^CD8^lo^, SP, Treg, and memory-like cells. These cell states or types were distinguished based on differentially-expressed gene (DEG) analysis, along with the expression patterns of CD4, CD8A, and human thymic developmental-stage markers [17] (Fig. 1B, Supplementary Table and Fig. 1A).

**Fig. 1:**
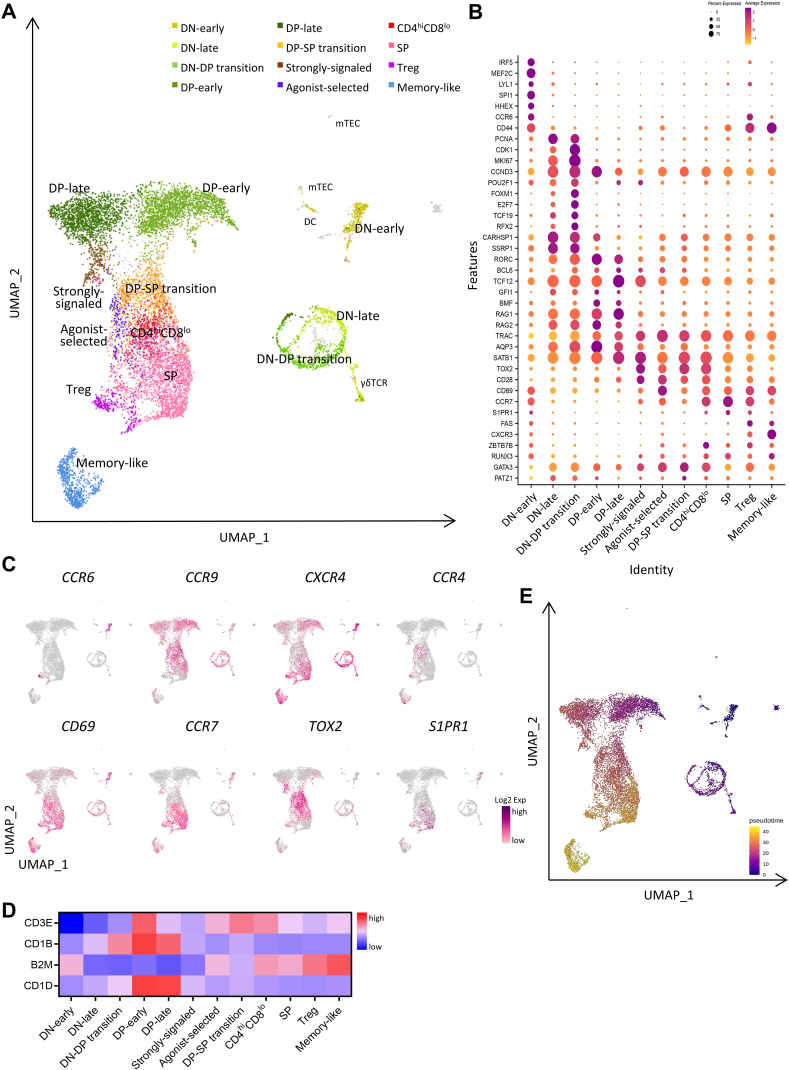
Single-cell RNA-sequencing analysis of total cynomolgus monkey thymocytes. (**A**) UMAP representing the cell types of total thymocytes of the cynomolgus monkey. More than 12 distinct cell types were defined according to the expression of thymic developmental-stage markers. Each color represents a defined cell type. (**B**) Dot plot showing the expression of the thymic developmental-stage markers of defined cell types. The color represents the average expression of marker genes, and the dot size indicates the percentage of cells expressing the marker genes presented in the subsets. (**C**) UMAP of *CCR6*, *CCR9*, *CXCR4*, *CCR4*, *CD69*, *CCR7*, *TOX2*, and *S1PR1* expression in total thymocytes. (**D**) Heatmap showing relative expression of *CD3E*, *CD1B*, *B2M*, and *CD1D* for defined cell types; Z-scores obtained by differentially-expressed gene analysis of analyzed cell types are shown. (**E**) UMAP displaying the pseudotime of the total thymocytes. UMAP, Uniform Maniform Approximation and Projection; DN, double-negative; DP, double-positive; SP, single-positive; Treg, regulatory T cell.

To validate T-cell developmental trajectories, we investigated the expression of genes that constitute hallmarks in T-cell differentiation. Such stage-specific genes include the proliferation-associated genes, *CDK1* and *MKI67*, the recombination-activating genes, *RAG1* and *RAG2*, and the stage-transition genes, *SATB1* and *TOX2* [17], which allowed us to distinguish the developmental status of each cell type. We observed that the developmental trajectory started from the DN stage and diverged at the DN–DP junction, which corresponds to the γδT cell differentiation. Subsequently, DN-late thymocytes were observed to transition to the DP-early stage.

We next investigated thymocyte trafficking to characterize how cells migrate to different thymic regions while undergoing positive and negative selection [21] (Fig. 1C). The DN-early stage was characterized by high *CCR6* expression, which is involved in the migration of uncommitted thymocytes to the thymus [22], along with the chemokine receptors, *CCR9* and *CCR7*. DN-late-stage thymocytes displayed high *CCR9* and CXCR4 expression [23,24] and appeared to migrate through their DN-early-stage counterparts to reach the DP stage. The DP–SP transition stage was characterized by chemokine markers associated with thymocyte migration to the thymic medulla, including high expression of CCR4 and CD69, along with low expression of CCR7 [[25], [26], [27]]. In this context, we focused on a distinct subset of DP thymocytes undergoing DP–SP transition, which were characterized by high TOX2 expression [17]. At this stage, immature DP thymocytes are assumed to follow conventional or agonist selection, depending on whether signaling through the TCR:pMHC involves low or high binding affinity. Regarding the conventional selection pathway, we identified a CD4^hi^CD8^lo^ intermediate DP stage characterized by high *ZBTB7B* and low *RUNX3* expression [15]. Subsequently, mature SP thymocytes that successfully underwent both a positive and a negative selection processes exhibited upregulated *S1PR1*, which is associated with exiting the thymus at the cortical-medullary junction [28].

*CD1B* expression, which is used as a distinguishing marker of thymocyte development stages [29], appeared to increase from the DN–DP transition stage onwards and decrease during thymocyte maturation. By contrast, *CD1D* expression was high during the DP-early stage, whereas *B2M* (MHC class-I) expression was the lowest during the DP-late stage. This inverse expression pattern of MHC class-I and *CD1D* at the immature DP state might represent a developmental opportunity to select for nonclassical MHC-restricted unconventional T cells, such as NKT [30]. Regarding the interactions among thymocytes, we found that the *CD3E*, *CD1B*, *B2M* (MHC class-I), and *CD1D* expression varied dynamically and sequentially as a function of T-cell developmental stages (Fig. 1D). Taken together, our DEG differentiation-stage analysis of signature markers and ligands fully reconstructed the T-cell development course, and pseudotime analysis showed consistent results of the T-cell development pathway (Fig. 1e).

### 3.2 Functional TCR recombination kinetics

To explore detailed features of T-cell developmental trajectories, we investigated the TCR recombination kinetics. To analyze patterns of TCR repertoire formation, we associated transcripts encoding TCR chains with our cell-type annotations (Fig. 2A). *TRGV* and *TRDV* were exclusively expressed at the DN-late stage, indicating their role in γδT cell selection. For TCRβ and pre-TCRα (*PTCRA*) [31], we observed expression of several *TRBV* genes that persisted from the initiation of the DN–DP transition to the DP-early stage, a kinetics that was recognized as beta selection. After beta selection, we also observed T-cell receptor alpha variable *(TRAV)* and *TRAC* gene upregulation (TCRα), which became steadily enriched from the DP-early to DP–SP transition stage.

**Fig. 2:**
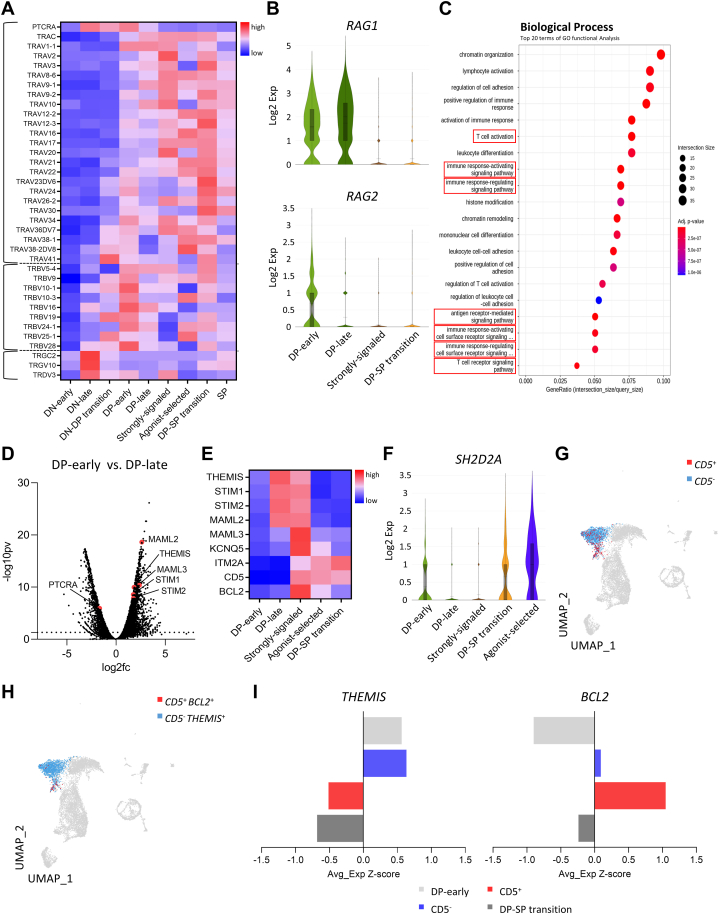
Dynamics of TCR recombination during T cell development. (**A**) Heatmap showing the relative TCR gene expression. Dotted lines distinguish among the genes encoding TCRα, TCRβ, and TCRγδ chains. (**B**) Violin plots showing *RAG1* and *RAG2* expression. (**C**) Dot plot showing the most significant GO terms of the DP-late cell state. GO processes are ordered according to their relative enrichment scores. Dot sizes represent the number of significant genes associated with the GO term. Dot colors represent the adjusted p-values. (**D**) Volcano plot showing differentially-expressed genes at the DP-early and DP-late cell states. Colored dots indicate the most representative marker genes among genes that are significantly different between the two populations. (**E**) Heatmap showing the relative expression of TCR signaling-associated genes. (**F**) Violin plot showing *SH2D2A* expression. (**G**) UMAP showing *CD5*^+^ and *CD5*^−^ cells in DP-late and strongly-signaled cell states. (**H**) UMAP showing *CD5*^+^*BCL2*^+^ and *CD5*^−^*BCL2*^+^ cells in DP-late and strongly-signaled cell states. (**I**) Comparison of Z-score-converted *THEMIS* and *BCL2* expression in *CD5*^+^ cells and *CD5*^−^ cells at DP-late and strongly-signaled cell states. All heatmaps show the Z-scores obtained by differentially-expressed gene analysis of the studied cell types. TCR, T-cell receptor; GO, gene ontology; UMAP, Uniform Maniform Approximation and Projection; DN, double-negative; DP, double-positive; SP, single-positive; -log10pv, -log10(p-value); log2fc, log2(fold change); Avg_Exp, Average expression.

Formation of the TCR repertoire by immature DP thymocytes was also supported by *RAG1* and *RAG2* expression. *RAG* expression has been reported to be transiently downregulated in cells that successfully rearrange TCRβ and differentiate along the αβ pathway [32]. Consistent with this observation, at the DP-early stage, both *RAG1* and *RAG2* genes exhibited a strong bias relative to the DP-late stage. However, we observed a marked depletion of *RAG2* expression at the DP-late stage (Fig. 2B). This indicates that the successful TCR arrangement appears to have been nearly completed at the DP-early state. Interestingly, *RAG2* expression reduction at the DP-late stage was strongly associated with successful TCR signaling, as revealed by Gene Ontology (GO) functional analysis (Fig. 2C). This is consistent with the observation that, following successful TCRα recombination and αβTCR assembly, *RAG* is permanently downregulated in DP thymocytes receiving TCR positive-selection signals [33]. Therefore, these results imply that the generation of diverse TCR repertoires dynamically occurs during the DN-late-to-DP–SP transition for thymic selection. Furthermore, it suggests that cells in both the DP-early and late stages acquire an important differentiation state among the thymic selection of αβT cells by means of TCR signaling.

### 3.3 Elimination of self-reactive thymocytes does not occur immediately

Following successful TCR rearrangement, a fraction of immature DP thymocytes are positively selected, but most are neglected or eliminated depending on the TCR-signaling strength [13,21]. Given that the monkey thymus tolerates the development of highly self-reactive T cells, we hypothesized that it may not immediately delete all immature DP thymocytes that receive strong TCR signaling. To test this hypothesis, we first analyzed the underlying gene signatures that provide evidence for strong TCR stimulation (Fig. 2D and E). In DP-late cells, high *MAML2* and *MAML3* expression, related to TCR-mediated Notch signaling [34,35], along with high *STIM1* and *STIM2* expression, related to calcium entry [36], were observed. This indicates that the DP-late state contains cells that have been exposed to strong TCR signaling. Additionally, DP-late cells exhibited gene signatures associated with high TCR affinity, as well as contrasting gene signatures. For instance, *THEMIS*, known to enhance the sensitivity to TCR signaling of thymocytes that would be neglected due to low affinity for self-antigens [37], showed high expression in DP-late cells. At this DP-late cell state, immature thymocytes are observed to acquire one of two fates: thymocytes that are not positively selected for self-ligand will either be neglected due to very low affinity or eliminated due to very high affinity.

Next, we focused on a cell population denoted as a strongly-signaled cell state, which leads to the DP–SP transition from the DP-late stage. This population, along with DP-late cells, showed significant *SH2D2A* downregulation, which is associated with sensitivity to cell death during thymic selection [38] (Fig. 2F and Supplementary Fig. 1B). That is, absence of *SH2D2A* expression indicates that thymocytes are less susceptible to activation-induced cell death caused by a sharp threshold of TCR avidity in the thymus [38]. This observation suggests that a fraction of immature DP thymocytes, excluded from positive selection, is likely to follow an alternative pathway instead of immediate deletion. We found that the expression of *CD5*, one of the earliest-characterized positive-selection markers, whose expression increases proportionally to the TCR signals [39], is gradually upregulated in this strongly-signaled population (Fig. 2G). We further analyzed the affinity-enhancement-associated gene, *THEMIS*, and the anti-apoptosis gene, *BCL2* [40], to determine whether all thymocytes that receive a low-affinity or strong TCR signal during the DP-late stage are immediately deleted or not. As expected, *THEMIS* expression was higher in *CD5*^−^ than in *CD5*^+^ cells, but *BCL2* expression was higher in *CD5*^+^ than in *CD5*^−^ thymocytes (Fig. 2h and i). These results indicate that a fraction of *CD5*^*+*^ immature thymocytes, which would be deleted by negative selection due to their high affinity for self-reactivity [39], may express *BCL2* to change their fate and follow alternative differentiation pathways.

### 3.4 Alternative agonist selection and conventional selection trajectories

**Fig. 3:**
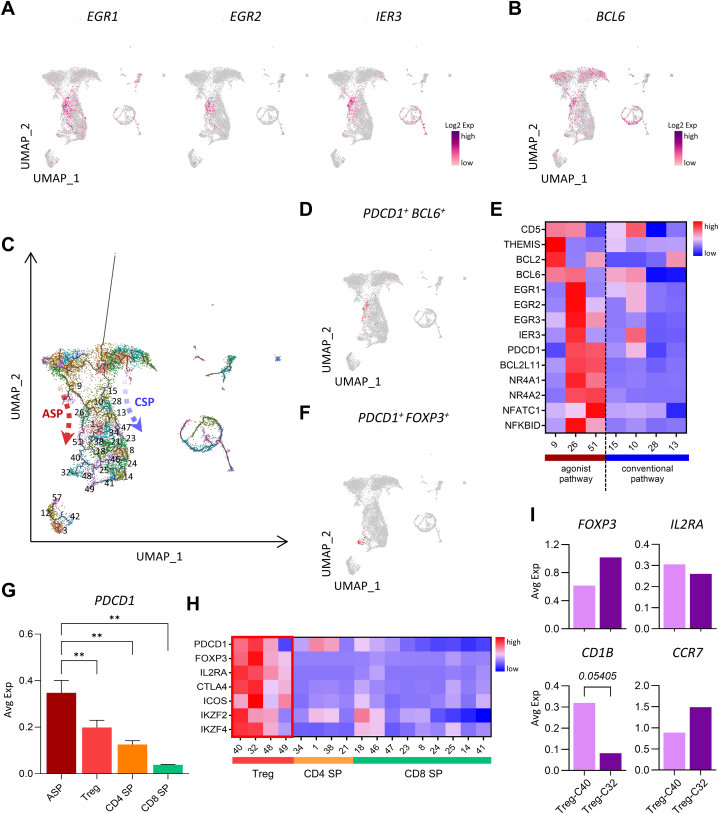
Alternative agonist selection and conventional selection trajectories. (**A**) UMAP of *EGR1*, *EGR2*, and *IER3* expression in total thymocytes. (**B**) UMAP of *BCL6* expression in total thymocytes. (**C**) UMAP of clusters of cynomolgus monkey thymocytes, detected using Louvain's algorithm. The black lines on the UMAP represent the trajectory graph. The two differently-colored arrows represent the two hypothetical developmental pathways. The number in UMAP indicates the cluster number. (**D**) UMAP showing the *PDCD1*^+^*BCL6*^+^ cells among the total thymocytes. (**E**) Heatmap showing the relative expression of survival-related markers and agonist selection-associated markers for clusters assigned to the two hypothetical developmental pathways. (**F**) UMAP showing the *PDCD1*^+^*FOXP3*^+^ cell population among the total thymocytes. (**G**) Comparison of the average *PDCD1* expression among clusters corresponding to different cell populations. (**H**) Heatmap showing the relative expression of Treg markers for clusters assigned to Treg, CD4 SP, and CD8 SP cell populations. (**I**) Graphs comparing the average expression *FOXP3*, *IL2RA*, *CD1B*, and *CCR7* for clusters 40 and 32, which correspond to the Treg cell population. All heatmaps show the Z-scores obtained by differentially-expressed gene analysis of the studied cell types. UMAP, Uniform Maniform Approximation and Projection; ASP, agonist selection pathway; CSP, conventional selection pathway; SP, single-positive; Treg, regulatory T cell; C40, cluster 40; C32, cluster 32; Avg Exp, Average expression; ∗∗, p-value <0.05.

Upon successful positive selection, immature thymocytes upregulate *CCR4* and *CCR7*, which facilitates their migration towards the medulla [41]. To elucidate the detailed thymocyte developmental pathways during the DP–SP transition stage, we examined survival-related genes. We identified separate immature thymocytes that shared EGR family gene (*EGR1*, *EGR2*) expression, which are involved in thymocyte survival during positive selection [42,43], as well as *IER3*, which inhibits apoptosis of activated T cells [44] (Fig. 3A). *BCL6* is induced by thymic TCR signaling in an ERK-dependent manner and provides survival signals by inhibiting pro-apoptotic gene expression during agonist selection [45]. Interestingly, we found that *BCL6* was distinctly expressed in a fraction of cells undergoing DP–SP transition (Fig. 3B). These observations suggest that immature thymocytes that receive strong TCR signals can diverge using alternative differentiation pathways.

Using the Louvain algorithm, we detected 63 clusters and thymocyte trajectories and further analyzed the gene signatures of each cluster, corresponding to the cell states and types of Fig. 1A (Supplementary Fig. 2A). We observed that *BCL6*^+^ cells were differentiated among strongly-signaled cells. To verify whether these cell populations undergo an alternative differentiation pathway, distinct from the conventional T-cell developmental pathway, we compared the two hypothetical developmental pathways (Fig. 3C). We first focused on clusters 9, 26, and 51, which are assumed to constitute an agonist selection pathway. In the coordinates assigned to *BCL6*^+^ cells, *PDCD1*, described as an agonist-selection marker [5,46], was very highly expressed compared with other clusters (Fig. 3D), which indicates that this population arises by agonist selection. In addition, we found that the expression of agonist selection-associated markers [47] such as *BCL2L11*, *NR4A1*, *NR4A2*, *NFATC1*, and *NFKBID* was pronounced along the same pathway (Fig. 3E and Supplementary Fig. 2B). By contrast, we observed that clusters 15, 10, 28, and 13 transitioned from the DP-early stage to the CD4^hi^CD8^lo^ intermediate population, in line with a conventional selection pathway. In this pathway, *BCL2*, *BCL6*, *CD5*, and EGR family gene expression levels were relatively lower than those of the agonist selection pathway genes. Based on these observations, we defined this maturation process as a conventional selection pathway. Our trajectory analysis showed that during positive selection, monkey T-cell development diverges toward either agonist selection or conventional selection.

We further studied the development of *FOXP3*^+^ Treg cells expressing the agonist-selection marker *PDCD1* (Fig. 3F). *PDCD1* expression was lower in Treg than in agonist-selected cell populations but higher than that of CD4 SP cell populations (Fig. 3G and Supplementary Fig. 2C). Recent studies have reported two types of Treg precursor populations associated with agonist selection [48]. To elucidate these subpopulations, we examined four clusters expressing *FOXP3* (40, 32, 48, and 49) (Fig. 3H). In clusters 40 and 32, the Treg-associated gene markers, *PDCD1*, *IL2RA*, *CTLA4* and *ICOS*, were expressed relatively higher than clusters 48 and 49. Moreover, cells in cluster 40 showed higher *IL2RA* and lower *FOXP3* expression than cluster 32 and could, therefore, be characterized as a differentiating Treg cell type. This was also consistent with *CD1B* and *CCR7* expression differences between the two clusters (Fig. 3I).

### 3.5 Memory-like cells characterized by CXCR3 and EOMES expression

**Fig. 4:**
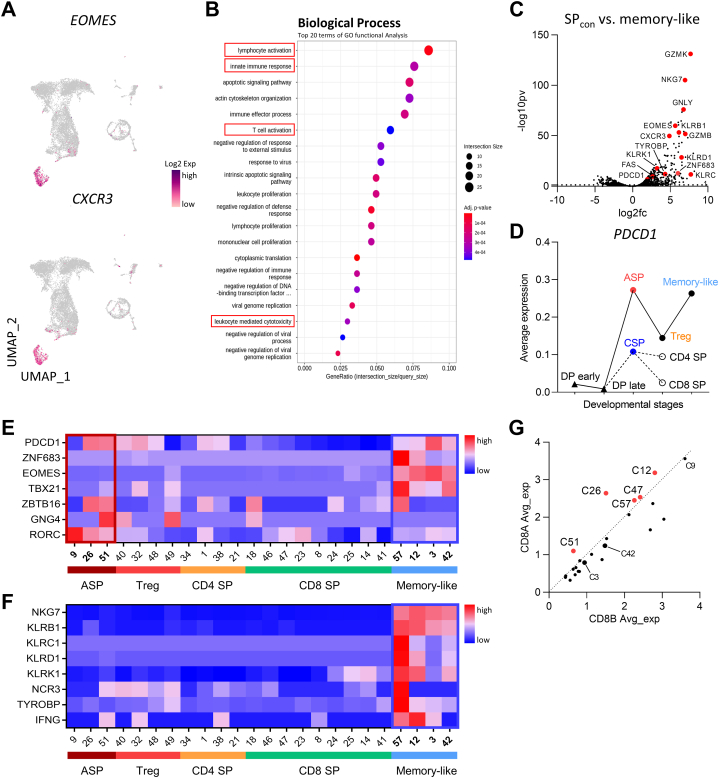
UMAP and DEG analysis of memory-like thymocytes. (**A**) UMAP of *CXCR3* and *EOMES* expression in total thymocytes. (**B**) Dot plot showing the most significant GO terms of the memory-like cells. GO processes are ordered according to their corresponding enrichment scores. Dot sizes represent the number of genes, significantly associated with each GO term. Dot colors represent the adjusted p-values. (**C**) Volcano plot showing differentially-expressed genes in SP_con_ (CD4^hi^CD8^lo^, Treg, and SP) and memory-like thymocytes. Colored dots indicate the most representative marker genes among genes that are significantly different between the two populations. (**D**) *PDCD1* expression levels according to developmental trajectory. Each dot is labeled according to cell population stage, and a cluster number is assigned to it. (**E-F**) Heatmap showing the relative expression of unconventional T-cell lineage-related gene markers for clusters corresponding to ASP, Treg, CD4 SP, CD8 SP, and memory-like cell populations. (E) *PDCD1* and transcription factors of unconventional T-cells. (F) innate-associated markers; Z-scores obtained by analyzing the differentially-expressed gene of the studied clusters are shown. (**G**) Scatterplot comparing *CD8A* and *CD8B* expression levels clusters corresponding to ASP, Treg, CD4 SP, CD8 SP, and memory-like cell populations. UMAP, Uniform Maniform Approximation and Projection; DEG, differentially-expressed genes; GO, gene ontology; ASP, agonist selection pathway; CSP, conventional selection pathway; -log10pv, -log10(p-value); log2fc, log2(fold change); SP_con_, conventional single-positive.

Among thymocytes reaching the maturation stage, we identified a large population of memory-like cells, characterized by the expression of *CXCR3* and *EOMES*, which constitute signature markers of the memory cell type (Fig. 4A). This observation is consistent with the report that >2 % of the thymocytes express EOMES in the monkey thymus [29]. GO functional analysis showed that this cell type was associated with activation, cytotoxicity, and innate immune response (Fig. 4B). DEG analysis of this cell type revealed high expression of effector markers such as *GZMK*, *NKG7*, and *GNLY*, as well as distinct gene signatures associated with of innate-like cell features, including *KLRB1*, *KLRC1*, *ZNF683*, and *TYROBP* expression (Fig. 4C).

To determine which developmental pathway is involved in the generation of the memory-like cell type, we compared the aforementioned cells with the DEGs of SP T cells that developed via conventional selection. We found that *PDCD1* was expressed at distinctly high levels in the memory-like cell type. Moreover, *PDCD1* expression in the memory-like cell type was higher than that in Tregs populations and showed a consistent pattern of high expression along the agonist selection pathway (Fig. 4D). Even among memory-like cells, *PDCD1* expression was predominantly high in the *EOMES* positive population (Supplementary Fig. 3A). Interestingly, this expression pattern between *PDCD1* and *EOMES* was consistent with our flow cytometric findings (Supplementary Fig. 3B). This observation indicates that many *EOMES* ^+^ memory-like cells of the monkey thymus highly express *PDCD1*, a marker of agonist selection [5,46] and suggest that these cells are strongly linked to the agonist selection pathway.

### 3.6 Unconventional T cells exhibiting innate-like characteristics within the memory-like populations

To explore the thymic development of key unconventional T cell subsets, including NKT cells, MAIT cells, and γδT cells, we examined their specific invariant TCR chains and hallmark gene expression (Supplementary Fig. 3C). We observed that γδT cells branched off at the DN-late stage, with *Vγ3*^+^ cells identified, and *Vγ10*^+^ γδT cells predominantly located in clusters 57 and 12. However, *Vδ2Vγ2*^+^ T cells, previously identified in rhesus macaques [49] or *Vδ2Vγ9*^+^ T cells observed in humans [50,51] were not detected in our dataset. Additionally, while MAIT cells are typically identified as *TRAV1-2* (Vα7.2) in humans [52], this marker was absent in our findings. Instead, we found the paralogous *TRAV1-1* gene, primarily expressed at the DP-stage (Fig. 2A). NKT cells, characterized by *TRAV10* (Vα24) expression, were observed in Memory-like cell populations. Notably, certain cells in cluster 57 co-expressed *KLRB1* and *ZBTB16*, markers associated with NKT cells, despite lacking co-expression of specific TCR genes. Furthermore, *TRAV10*^+^ cells in clusters 12 and 3 also co-expressed *KLRB1* (Supplementary Figs. 3D and 3E). Although our data did not fully define MAIT, γδT, and NKT cells expressing their respective signature genes (e.g., *ZBTB16*, *KLRB1*, and *IL18R*), these unconventional T cell populations exhibited overlapping gene signatures and were predominantly distributed within memory-like clusters. Recently, the thymic development of various unconventional T-cell lineages has been described in humans, including *GNG4*^+^*CD8αα*^+^ T(I) cells and *ZNF683*^+^*CD8αα*^+^ T(II) cells [17,46,47]. To determine if similar subsets exist among monkey thymocytes, we explored genes whose expression constitutes hallmarks of unconventional T-cell lineage development. *GNG4*^+^ cells were identified only in small numbers in the agonist-selected cell populations (cluster 51), with a few *GNG4*^+^ cells also distributed across other clusters, including clusters 49, 40, and 18. In contrast, *ZNF683*^+^ cells were distinctly found in the memory-like cell populations (cluster 57 and 12) (Fig. 4E). In terms of T-cell differentiation, *GNG4*^+^ cells appeared to emerge before the development of *ZNF683*^+^ cells. Overall, cells in clusters 57 and 12 were characterized by prominent expression of *KLRC1*, *KLRD1*, *KLRK1*, *NCR3*, and *TYROBP*, indicating features of innate-like cells (Fig. 4F).

Subsequently, we examined the expression patterns of *CD8αα* and *PDCD1* to evaluate the association of these unconventional T cells with agonist selection. *CD8A* expression was higher relative to *CD8B* in the agonist-selected cell populations (cluster 26 and 51) and in unconventional T cells (cluster 57 and 12) within memory-like cell populations (Fig. 4G). This suggests that these clusters are likely to express CD8αα homodimer, even based on transcript data. Together, these results suggest that unconventional T-cell lineages of the human thymus are conserved in the monkey thymus and can similarly develop and mature by means of agonist selection.

## 4 Discussion

In this study, we reconstructed the differentiation trajectories of both conventional and unconventional T-cell lineages of the cynomolgus monkey thymus. During thymic development, T cells undergo various fate decisions, including positive selection, neglect, negative selection, and agonist selection, depending on their TCR specificity [13]. Our findings demonstrated that immature DP thymocytes can adopt an unconventional T cell fate trajectory instead of clonal elimination via negative selection, even after receiving a strong TCR signal.

Our objective was to distinguish between uncommitted cells directed for agonist selection and those following the conventional cell lineage fate. We studied the transcriptional profiling of DP thymocytes exhibiting strong TCR and calcium signaling (*STIM1*, *STIM2*) [36], along with selective survival signaling (*NFAT5*) [53]. Noteworthily, high expression of *BCL2* [40] and *BCL6* [45,54] among immature DP thymocytes suggested that not all self-reactive thymocytes that received strong TCR signals are eliminated by means of negative selection. In this context, the relatively high *SH2D2A* expression in the agonist-selected population suggests that this population has an inherently high level of self-reactivity, despite evading negative selection. This effect of elevated *SH2D2A* may be counteracted by the increased expression of *BCL2*, *EGR* family, and *IER3*. This observation is supported by studies indicating that high levels of the anti-apoptotic protein, Bcl-2, can rescue apoptotic thymocytes from death both in vitro and in vivo [55]. Moreover, it aligns with reports indicating that upregulation of *BCL6* by TCR signaling in DP thymocytes is crucial for the survival of agonist-selected IEL-lineage progenitor cells [45]. Therefore, our findings indicate that despite the strong TCR-selection signals, a thymocyte fraction expressing *BCL2* and *BCL6* manages to survive and is rescued from apoptosis.

Recent research suggests that the agonist-selection checkpoint precedes the conventional selection in the human thymus [5]. TCR stimulation of immature thymocytes has been proposed to fix early *TRAV* and T-cell receptor alpha joining (*TRAJ*) rearrangements of the TCR repertoire, leading to the generation of an innate subset [5]. This implies that self-reactive T cells may result from an instructed program rather than simply from escaping negative selection [5,13]. In other words, self-reactive DP thymocytes are not immediately eliminated but might edit their TCRs to avoid death signals. According to a hypothetical model of human thymic agonist selection [47], cells of the agonist-selection lineage undergo branching at the corticomedullary junction, to which *XCR1*-expressing conventional dendritic cells (DC1) are recruited. Consequently, the agonist-selection lineages are thought to express *CCR4* and *CD4*0LG to interact with DC1 cells. Although our data did not allow us to confirm that the corticomedullary junction is the checkpoint location, given the expression of *CCR4* and *CD4*0LG in our study, agonist selection appears to occur prior to lineage commitment to SP T cell fate via conventional selection.

A recent study highlighted that among CD8 T-cell precursors, cells with a memory phenotype display *EOMES* upregulation during thymic maturation before a full memory phenotype is induced [56]. This is believed to be a unique program triggered by self-ligand recognition. Considering that the phenotype and function of agonist-selected cells may depend on repeated encounters with self-antigens, agonist selection could resemble a chronic immune response rather than a developmental transition [13]. In this context, our observation that a large cell population expressed memory markers such as *EOMES* and *CXCR3* indicates the association of these markers with agonist selection. Furthermore, *PD-1* expression on peripheral unconventional T cells serves as a footprint of TCR stimulation during agonist selection in the thymus [46]. This aligns with our findings that *PDCD1* expression remained consistently high, even within memory-like cell populations along the agonist-selection pathway. Consequently, these findings suggest a strong association between *EOMES* ^+^ effector/memory cell development in the monkey thymus and *PDCD1* expression.

Recent studies have established that CD3^+^ DP postnatal thymic cells diverge into two main pathways: the unconventional pathway, which includes *GNG4*^+^*CD8αα*^+^ T(I), *ZNF683*^+^*CD8αα*^+^ T(II), and TCRγδ cells, and the conventional pathway, consisting of CD4 and CD8 SP T cells [46]. Specifically, ZNF683^+^CD8αα^+^ T cells have been identified as CD10^+^PD-1^+^TCRαβ^+^ innate-like T-cell precursors [46]. This CD10^+^PD-1^+^TCRαβ^+^ differentiation pathway diverges from the one specifying the conventional SP T-cell lineages at the early DP stage and is characterized by the developmental-hallmark transcription factors *ZNF683* and *IKZF2* [46]. Additionally, *CD8αα* expression in thymocytes denotes a distinct precursor subset, and CD8αα^+^ T cells are known to exhibit a typical innate functional effector behavior [5]. Consistent with this, our transcriptome profile showed that *ZNF683*^+^ cell lineages expressed *CD8αα*, and the corresponding cell population exhibited elevated innate function-associated signature markers. Thus, along with *PD-1*, *CD8αα* expression could be considered a crucial hallmark of the differentiation pathway and functional characteristics of unconventional T cells undergoing agonist selection.

NKG2A/C^+^CD8^+^ T cells have been reported to be macaque innate-memory cells [57], whose expansion is induced by IL-15 and may contribute to controlling viral infection [58]. Polyclonal CD8^+^ T cells, characterized by a marked innate/memory phenotype, high *EOMES* expression, and the ability to rapidly generate IFN-γ without prior antigen exposure, have been described in mice [59], as well as in human adults and cord blood [60]. We aimed to further identify the thymic progenitors of unconventional T-cell lineages, such as the Eomes^+^NKG2A^+^ CD8^+^ T [57] and Nkp30^+^ CD8^+^ T [61] subsets observed in the periphery. However, our transcriptome profiling presented limitations in identifying progenitors of these innate-like cells. This is because data interpretation was complicated by the fact that cells expressing the *KLRC1* (NKG2A) and *NCR3* (Nkp30) markers in the monkey thymus displayed similar transcriptional profiles with *ZBTB16*^+^ cell populations. To overcome these limitations, further studies with more thymic T cells and non-T cells are required.

The absence of validation of the scRNA-seq data with complementary flow cytometry evidence remains a limitation of our study. This study has limitations, including the use of data from a single individual, which may affect representativeness. The cell isolation process may also impact the detection of rare populations. Future multi-omics studies across multiple cynomolgus monkeys would help address these issues and provide a deeper understanding of T cell development.

## 5 Conclusion

This study revealed the thymocyte heterogeneity and reconstructed T-cell development through single-cell transcriptome analysis in the monkey thymus. Furthermore, our findings provide insight into alternative selection pathways that direct self-reactive thymocytes to acquire unconventional T-cell lineage fates. These findings provide valuable insights into T cell immunity in cynomolgus monkeys, a key nonhuman primate model widely used in infectious disease and vaccine research. In particular, this study enhances our understanding of unconventional T cells, informing future investigations and improving the interpretation of immune response in this model.

## CRediT authorship contribution statement

**Sung Min Choi:** Writing – original draft, Formal analysis, Data curation, Conceptualization. **Kyeong Cheon Jung:** Writing – review & editing, Supervision, Conceptualization. **Jae Il Lee:** Writing – review & editing, Writing – original draft, Supervision, Conceptualization.

## Ethics statement

The study was approved by the local Institutional Animal Care and Use Committee (IACUC) of Seoul National University Hospital (IACUC number: 21-0297-C1A0). All experiments were performed in accordance with the relevant guidelines and regulations and the ARRIVE guidelines.

## Data availability

All data generated or analyzed during this study are included in this published article and its supplementary information files. All single-cell RNA-sequencing data are deposited in the Gene Expression Omnibus (GEO) under the accession number GSE267379.

## Funding

This study was supported by a grant from the 10.13039/501100003725National Research Foundation (NRF) of Korea, funded by the Korean 10.13039/501100014188Ministry of Science and ICT (grant number: NRF-2021R1F1A1045856).

## Declaration of competing interest

The authors declare that they have no known competing financial interests or personal relationships that could have appeared to influence the work reported in this paper.

## Figures and Tables

**figs1:**
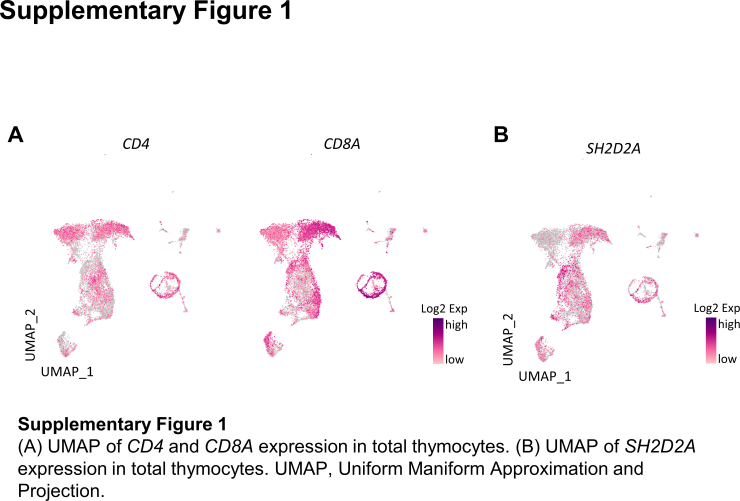


**figs2:**
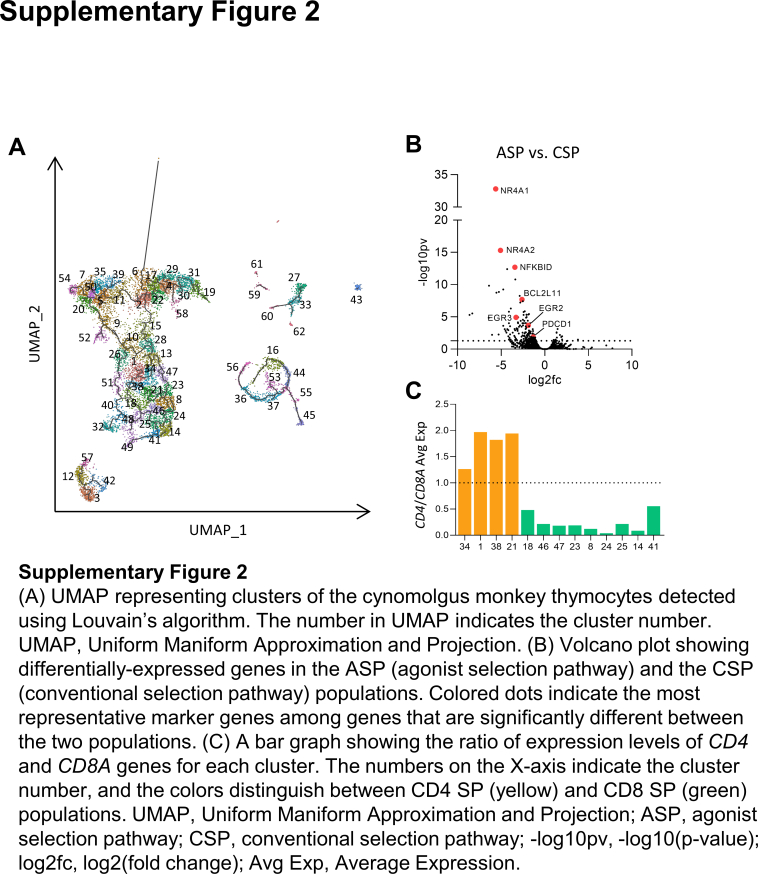


**figs3:**