# Supplementary material for: Developmental trajectory of unconventional T cells of the cynomolgus macaque thymus
Source: Heliyon. 2024 Oct 23;10(21):e39736. doi: 10.1016/j.heliyon.2024.e39736 (PMC11543906; doi:10.1016/j.heliyon.2024.e39736)
Supplement: Multimedia component 1 [file mmc1.docx]

**Supplementary Table**. A table summarizing the key genes that are specifically highly expressed among the differentially expressed genes in each thymocyte population

| Populations | Differentially Expressed Genes |
| --- | --- |
| DN-early | *IRF5, MEF2C, LYL1, SPI1, CCR6* |
| DN-late | *PCNA, CDK1, CCR9, CXCR4* |
| DN-DP transition | *CDK1, MKI67, FOXM1, E2F7, CD1B* |
| DP-early | *RORC, RAG1, RAG2, AQP3* |
| DP-late | *TCF12, RAG1, SATB1, MAML2, MAML3* |
| Strongly-signaled | *CD5, BCL2, THEMIS, MAML3, KCNQ5* |
| Agonist-selected | *PDCD1, NR4A1, BCL2L11, EGR1, IER3* |
| DP-SP transition | *CCR4, CD69, TOX2, GATA3, PATZ1* |
| CD4^hi^CD8^lo^ | *ZBTB7B, CD4, TOX2, TRAC* |
| SP | *CD4/CD8A, CCR7, S1PR1* |
| Treg | *FOXP3, IL2RA, CTLA4, IKZF2, IKZF4* |
| Memory-like | *EOMES, CXCR3, GZMK, NKG7, KLRB1* |
